# Application of machine learning algorithms in predicting HIV infection among men who have sex with men: Model development and validation

**DOI:** 10.3389/fpubh.2022.967681

**Published:** 2022-08-25

**Authors:** Jiajin He, Jinhua Li, Siqing Jiang, Wei Cheng, Jun Jiang, Yun Xu, Jiezhe Yang, Xin Zhou, Chengliang Chai, Chao Wu

**Affiliations:** ^1^School of Public Health, Zhejiang University School of Medicine, Hangzhou, China; ^2^School of Software Technology, Zhejiang University, Ningbo, China; ^3^Zhejiang Provincial Center for Disease Control and Prevention, Hangzhou, China; ^4^School of Public Affairs, Zhejiang University, Hangzhou, China

**Keywords:** machine learning, HIV, MSM, prediction, models

## Abstract

**Background:**

Continuously growing of HIV incidence among men who have sex with men (MSM), as well as the low rate of HIV testing of MSM in China, demonstrates a need for innovative strategies to improve the implementation of HIV prevention. The use of machine learning algorithms is an increasing tendency in disease diagnosis prediction. We aimed to develop and validate machine learning models in predicting HIV infection among MSM that can identify individuals at increased risk of HIV acquisition for transmission-reduction interventions.

**Methods:**

We extracted data from MSM sentinel surveillance in Zhejiang province from 2018 to 2020. Univariate logistic regression was used to select significant variables in 2018–2019 data (*P* < 0.05). After data processing and feature selection, we divided the model development data into two groups by stratified random sampling: training data (70%) and testing data (30%). The Synthetic Minority Oversampling Technique (SMOTE) was applied to solve the problem of unbalanced data. The evaluation metrics of model performance were comprised of accuracy, precision, recall, F-measure, and the area under the receiver operating characteristic curve (AUC). Then, we explored three commonly-used machine learning algorithms to compare with logistic regression (LR), including decision tree (DT), support vector machines (SVM), and random forest (RF). Finally, the four models were validated prospectively with 2020 data from Zhejiang province.

**Results:**

A total of 6,346 MSM were included in model development data, 372 of whom were diagnosed with HIV. In feature selection, 12 variables were selected as model predicting indicators. Compared with LR, the algorithms of DT, SVM, and RF improved the classification prediction performance in SMOTE-processed data, with the AUC of 0.778, 0.856, 0.887, and 0.942, respectively. RF was the best-performing algorithm (accuracy = 0.871, precision = 0.960, recall = 0.775, F-measure = 0.858, and AUC = 0.942). And the RF model still performed well on prospective validation (AUC = 0.846).

**Conclusion:**

Machine learning models are substantially better than conventional LR model and RF should be considered in prediction tools of HIV infection in Chinese MSM. Further studies are needed to optimize and promote these algorithms and evaluate their impact on HIV prevention of MSM.

## Introduction

Acquired immune deficiency syndrome (AIDS) caused by the human immunodeficiency virus (HIV) is a global health crisis, which destroys the human immune system and gives rise to a variety of opportunistic infections and death (1, 2). Men who have sex with men (MSM) are one of the highest-risk populations for HIV acquisition because of their tendency to have multiple sexual partners and unprotected anal intercourse (3). Therefore, this group has now received special attention from society.

In China, an increasing body of evidence from different periods and locations has suggested that MSM play an important role in the HIV epidemic. A large-scale systematic analysis, in which data were extracted from 355 cross-sectional studies covered 59 cities from 2001 to 2018, found that the overall national prevalence of HIV among MSM was estimated to be 5.7% (95% CI: 5.4–6.1%), exceeding the WHO 5% AIDS epidemic warning threshold (4, 5). And two reports by the Chinese Center for Disease Control and Prevention (CDC) showed that the proportion of newly identified HIV/AIDS cases due to male-to-male intercourse has increased rapidly, from 13.7% in 2011 to 25.5% in 2017 (6, 7). To improve the status quo, the Chinese government has taken actions to promote HIV testing by MSM, such as Pilot Program for HIV/AIDS Comprehensive Intervention, peer education, and free HIV voluntary counseling and testing (8). However, only 47% of Chinese MSM had ever tested for HIV in 2016, and only 38% had tested for HIV in the last 12 months (9). This is still far behind the target of the Joint United Nations Programme on HIV/AIDS (UNAIDS) for 90% testing among infected individuals (10). It is urgent to develop a reliable model to identify early infected MSM in order to reduce the transmission of virus in this group, which can make up for the defect of incomplete coverage of HIV testing to a certain extent.

Previous studies have used logistic regression or Cox proportional hazards regression models to establish the prediction tool of HIV infection among MSM, but performance is not great due to the problems of data structure which are often non-linear, abnormal, and heterogeneous (11–14). Compared to the above traditional models, the machine learning algorithm provides a new method to construct models, since it can balance the deviation and variance of data (15). Nowadays, machine learning has been widely applied in the medical field, mainly reflected in medical auxiliary diagnosis and classification prediction, such as image-based cancer diagnostics (16, 17). However, machine learning algorithms have not been used to predict HIV infection among MSM, especially in China. In the present study, we focused on the Chinese MSM population and aimed to develop prediction models for HIV acquisition using logistic regression and several machine learning approaches. The processing of imbalance data by SMOTE before modeling is different from previous related studies. The predictive performance of these models is tested to determine the one that can most accurately identify high-risk MSM individuals with HIV, thus providing a basis for timely intervention and treatment of this population.

## Materials and methods

### Study population and data collection

MSM Sentinel Surveillance is a national government public health activity. The survey subjects were recruited by Non-Governmental Organizations and local CDC using snowball sampling at MSM event venues or online, with one-on-one questionnaires administered by trained enumerators and 5 ml of venous blood collected. Verbal consent was obtained from all study participants before survey and collection of specimens. Therefore, institutional review board approval was not required for analysis using sentinel surveillance data in China.

In this study, the cross-sectional data was derived from the questionnaire records which were collected from the MSM sentinel surveillance in Zhejiang province between 2018 and 2020. We included MSM that: (i) had oral or anal sex with other men within the past year, (ii) currently resided in Zhejiang Province, and (iii) were aged ≥ 15 years at the time of the survey. We excluded MSM that: (i) had already tested positive for HIV every year, (ii) disagreed to be blood collected. The main content of the questionnaire included five parts: general demographic information, AIDS-related knowledge, the occurrence of sexual behaviors, prevention services, and HIV antibodies testing. HIV antibodies testing used ELISA reagents for initial screening and retesting, and Western Blot was used for confirmatory testing when the results of both tests were positive.

### Data processing

Some samples may exist with missed or abnormal values, so we performed data cleaning to delete them. In addition, we also performed data transformation on the several features: “age” was divided into four classes according to Chinese age group classification (<18, 18–40, 41–65, >65); as for “AIDS-related knowledge”, if the results of 8 questions turn out to be all right, we would give a value of 1, otherwise, value of 0 will be given; “time of last HIV test” can be converted to dichotomous variables that whether had been tested for HIV in the past year or not. After data processing, continuous variables were presented as mean ± standard deviation or median [interquartile range (IQR)], and categorical variables were presented as the frequency number (percentage).

### Feature selection

The purpose of the feature selection was to eliminate redundant and irrelevant variables. Potential features can be selected by traditional statistical methods (15). We applied the filter method of univariate logistic regression to choose the feature subsets in which the independent variables are correlated with the dependent variable in the original data structure. Variables with statistical significance (*p*-value < 0.05) were selected as predicting features. As an estimate of effect size and variability, we have reported the odds ratio (OR) with a 95% confidence interval (CI).

### Data balancing

As the proportion of MSM infected with HIV was imbalanced in this study, we can apply resampling method to handle the disproportionate ratio of observations in each class. The technology of resampling consisted of random under-sampling (RUS) and random over-sampling (ROS). However, RUS removed a number of samples of the majority class so that lost some information. In our experiments, we performed the Synthetic Minority Over-sampling Technique (SMOTE) to balance data (18). SMOTE could generate synthetic data to increase the number in the smaller class by using the nearest neighbor's algorithm (19).

### Model establishing

We explored three classic machine learning algorithms for predicting HIV infection in MSM compared with Logistic Regression (LR), including Decision Tree (DT), Support Vector Machines (SVM), and Random Forest (RF). These algorithms are widely used for classification problems, and each has its unique features and advantages. LR is a generalized linear regression model that can apply a non-linear sigmoid function to predict the results of two sets of classifications through a series of continuous or categorical variables (20). DT uses tree structure to classify data in a hierarchical fashion and is recommended for problems in which input variables are discrete and final classification is binary (21). SVM employs the “max-margin principle” to create a decision boundary that is as far as possible from the closest data points from each of the classes (22). RF is an ensemble version of decision tree by aggregating predictions from multiple decision trees for a better model, which is more robust against overfitting (23).

### Model evaluation

Several standard indicators can be adopted to evaluate models' performance: accuracy, precision, recall, F-measure, and the area under the receiver operating characteristic curve (AUC). The result of a classification job can be classified into four categories in a confusion matrix that can explain the evaluation metrics for better understanding (24), and the tabular form of output is shown in Table 1. Relative concepts were shown as follows (15, 20).

**Table 1:** The confusion matrix.

	**Positive actual case**	**Negative actual case**
Positive prediction	True positive (TP)	False positive (FP)
Negative prediction	False negative (FN)	True negative (TN)

Accuracy measures the ratio of correct classification.


$$\begin{array}{l} \text{Accuracy }=\frac{\text{TP}+\text{TN}}{\text{TP}+\text{TN}+\text{FP}+\text{FN}} \end{array}$$

Precision means the proportion of positive prediction that are positive actual cases.


$$\begin{array}{l} \text{Precision }=\frac{\text{TP}}{\text{TP}+\text{FP}} \end{array}$$

Recall represents the fraction of positive actual cases that are correctly predicted.


$$\begin{array}{l} \text{Recall }=\frac{\text{TP}}{\text{TP}+\text{FN}} \end{array}$$

F-measure is the weighted harmonic mean of precision and recall. The higher the F-measure, the better predictive power of the model.


$$\begin{array}{l} \text{F}-\text{measure}=\frac{2\times \text{ Precision }\times \text{ Recall }}{\text{ Precision }+\text{ Recall}} \end{array}$$

AUC is a measure of the discriminative ability of prediction models which represents the area under the receiver operating characteristic curve (ROC). The vertical coordinate of the ROC curve is the true positive rate, and the horizontal coordinate of the ROC curve is the false positive rate. The value of AUC is between 0.5 and 1, and the closer to 1 indicates the better performance of the model (25).

**Figure 1:**
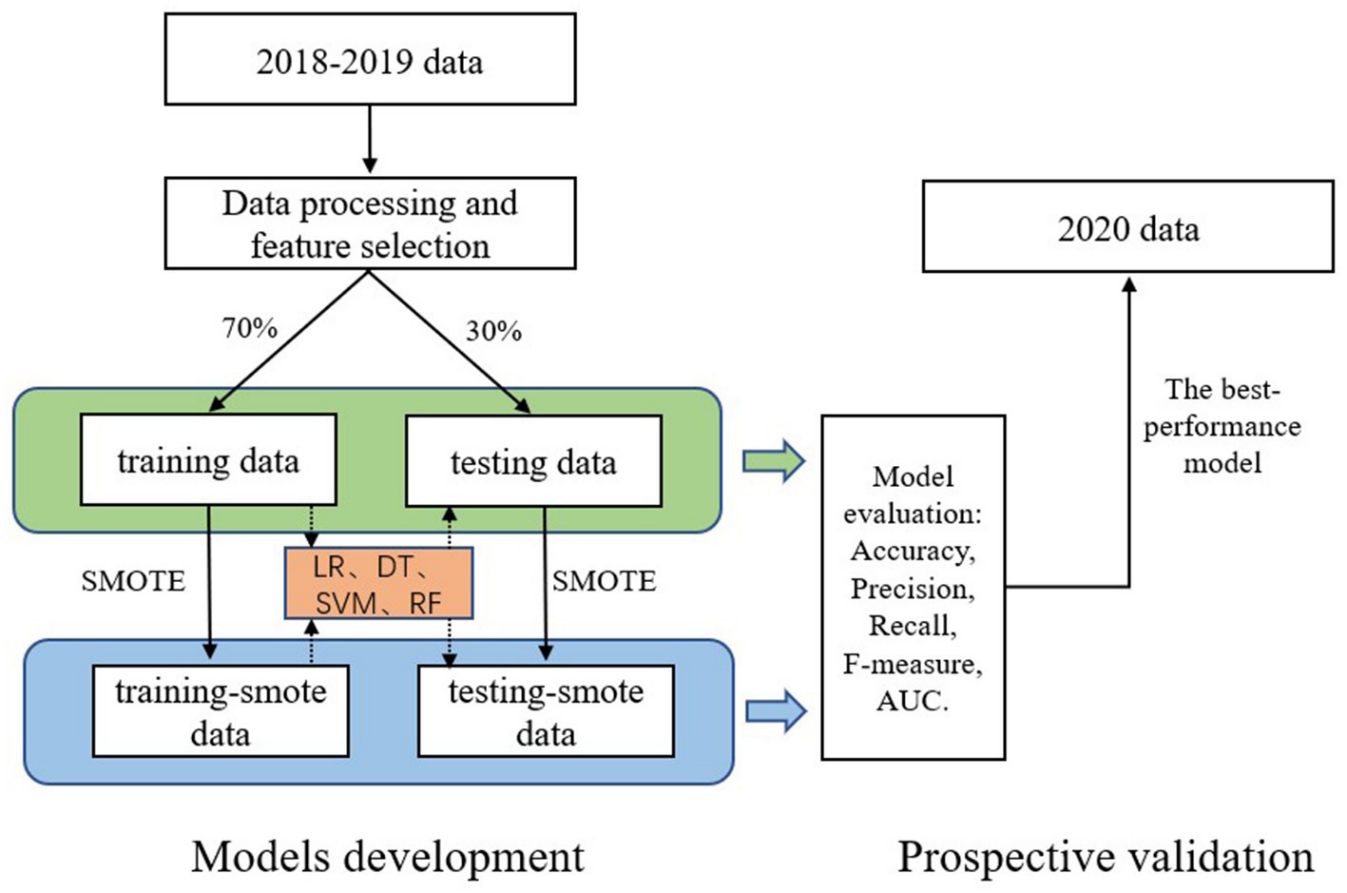
Flow chart of models development and prospective validation. LR, logistic regression; DT, decision tree; SVM, support vector machines; RF, random forest.

In this study, we built our models according to the Transparent Reporting of a multivariable prediction model for Individual Prognosis or Diagnosis statement for prediction models (26) (see Figure 1 for flow chart). After data processing and feature selection, we divided the 2018–2019 data into two groups by stratified random sampling based on the layer of “HIV-positive or negative”: training data (70%) and testing data (30%). Secondly, both training data and testing data were balanced by SMOTE. Then, the hyperparameters optimization of each model was obtained by grid-search and 5-fold cross-validation on the training data (see Supplementary Table 1). Finally, these models were verified on the testing data. We also conducted prospective validation of four models in the 2020 data. All data analyses were carried out with R software 4.1.2 version and Python software 3.9 version.

## Results

### Demographic characteristics

After data processing in 2018–2019 data, we included 6,346 MSM into this study, 372 of whom were infected with HIV (5.86%). The median age of them was 30.0 (IQR: 25.0–39.0) years, with 72 (1.13%) younger than 18 years, 4,993 (78.68%) aged 18–40 years, 1,245 (19.62%) aged 41–65 years, and 36 (0.57%) older than 65 years. And among them, 3,821 (60.21%) were unmarried; 6,557 (98.12%) identified as the Han ethnicity; 3,836 (60.45%) were census register of Zhejiang; 2,207 (34.78%) had obtained a college degree or above of education background.

### Feature selection

Univariate logistic regression analysis was performed to search the possible predictors and their associations with HIV infection. Descriptive summaries were shown in Table 2. Of all 27 potential predictors, age, marital status, census register, ethnicity, years of living in Zhejiang, AIDS-related knowledge, Condom use in the latest homosexual sex, frequency of condom use during homosexual sex in the past 6 months, diagnosed with sexually transmitted diseases, condom promotion/AIDS counseling and testing, AIDS peer education and HIV test in the past year were associated with HIV acquisition (*P* < 0.05), suggesting that these 12 variables can be used as predicting features.

**Table 2:** Basic characteristics of variables in both 2018–2019 and 2020 MSM and univariate associations of potential predictors with HIV infection in 2018–2019 MSM.

**Variables**	**2018–2019 MSM**		**Odd ratio (95% confidence interval)**	***P*-value**	**2020 MSM**	
	**No-HIV**	**HIV**			**No-HIV**	**HIV**
	***N* = 5,974**	***N* = 372**			***N* = 3,219**	***N* = 145**
	**(94.14%)**	**(5.86%)**			**(95.72%)**	**(4.28%)**
**Age (years)**						
<18	66	6	Ref.		27	2
18–40	4,720	273	0.64 (0.27, 1.48)	0.294	2,470	108
41–65	1,161	84	0.80 (0.34, 1.89)	0.605	698	34
>65	27	9	3.67 (1.19, 11.30)	0.024	24	1
**Marital status**						
Unmarried	3,574	247	Ref.		1,974	92
Married	1,990	102	0.74 (0.59, 0.94)	0.013	992	44
Cohabiting	49	2	0.59 (0.14, 2.44)	0.467	21	0
Divorced or widowed	361	21	0.84 (0.53, 1.33)	0.461	232	9
**Census register of Zhejiang**						
No	2,289	221	Ref.		1,260	86
Yes	3,685	151	0.42 (0.34, 0.53)	<0.001	1,929	59
**Ethnicity**						
Han	5,878	349	Ref.		3,161	139
Others	96	23	4.04 (2.53, 6.44)	<0.001	58	6
**Years of living in Zhejiang**						
<3 months	280	28	Ref.		155	10
3–6 months	269	16	0.59 (0.31, 1.12)	0.110	107	9
7–12 months	467	24	0.51 (0.29, 0.90)	0.021	197	4
1–2 years	849	51	0.60 (0.37, 0.97)	0.037	633	21
>2 years	4,109	253	0.62 (0.41, 0.93)	0.020	2,127	101
**Education background**						
Illiteracy	35	4	Ref.		6	0
Primary school	250	18	0.63 (0.20, 1.97)	0.427	107	8
Junior high school	1,553	121	0.68 (0.24, 1.95)	0.475	838	41
Senior high school	2,047	111	0.47 (0.16, 1.36)	0.165	1,153	38
College degree or above	2,089	118	0.49 (0.17, 1.41)	0.189	1,115	58
**Sexual orientation**						
Homosexuality	3,994	249	Ref.		2,289	102
Heterosexuality	48	2	0.67 (0.16, 2.76)	0.578	36	1
Bisexuality	1,743	109	1.00 (0.79, 1.27)	0.979	774	40
Unascertained	189	12	1.02 (0.17, 1.41)	0.952	120	2
**Places of seeking sex partners**						
Bar/dance hall	339	13	Ref.		260	0
Tearoom/clubhouse	157	10	1.66 (0.71, 3.87)	0.240	143	8
Public bath	329	20	1.58 (0.78, 3.24)	0.206	175	5
Park	257	6	0.61 (0.23, 1.62)	0.321	72	1
Internet	4,761	315	1.73 (0.98, 3.04)	0.059	2,527	179
Others	131	8	1.59 (0.65, 3.93)	0.313	42	2
**AIDS-related knowledge**						
No	2,529	203	Ref.		1,143	71
Yes	3,445	169	0.61 (0.50, 0.75)	<0.001	2,076	74
**Homosexual sex in the past week**						
No	3,062	209	Ref.		1,630	91
**Yes**	2,912	163	0.82 (0.66, 1.01)	0.065	1,589	54
**Condom use in the latest homosexual anal sex**						
No	1,072	158	Ref.		363	39
Yes	4,902	214	0.30 (0.24, 0.37)	<0.001	2,856	106
**Frequency of condom use during homosexual sex in the past 6 months**						
Never	249	39	Ref.		105	10
Sometimes	2,430	233	0.61 (0.43, 0.88)	<0.001	817	82
Every time	3,295	100	0.19 (0.13, 0.29)	<0.001	2,297	53
**Commercial sex in the past 6 months**						
No	5,730	362	Ref.		3,105	139
Yes	244	10	0.65 (0.34, 1.23)	0.186	114	6
**Heterosexual sex in the past 6 months**						
No	4,602	298	Ref.		2,636	122
Yes	1,372	74	0.83 (0.64, 1.08)	0.171	583	23
**Drug-taking**						
No	5,912	366	Ref.		3,213	144
Yes	62	6	1.56 (0.67, 3.64)	0.300	6	1
**Diagnosed with sexually transmitted diseases**						
No	5,725	350	Ref.		3,116	137
Yes	249	22	1.45 (1.02, 2.26)	0.008	103	8
**Condom promotion/AIDS counseling and testing**						
No	1,406	124	Ref.		572	45
Yes	4,514	248	0.65 (0.52, 0.81)	<0.001	2,647	100
**Community drug maintenance therapy/cleaning needle provision**						
No	5,585	344	Ref.		3,038	138
Yes	389	28	1.17 (0.78, 1.74)	0.444	181	7
**AIDS peer education**						
No	3,059	225	Ref.		1,697	77
Yes	2,915	147	0.69 (0.55, 0.85)	0.001	1,522	68
**HIV test in the past year**						
No	2,691	212	Ref.		1,278	67
Yes	3,283	160	0.62 (0.50, 0.76)	<0.001	1,941	78

### Performance comparison of the models

After data processing and feature extraction, there are three stages in our approach of model construction. The first stage is stratified random sampling on the whole model development dataset: training data (*n* = 4,442) and testing data (*n* = 1,904). We also implemented the resampling techniques of SMOTE in the original training data and testing data separately: training-smote data (*n* = 8,364) and testing-smote data (*n* = 3,584). Details showed in Table 3. Take the original data and SMOTE-processed data as input for the next stage.

**Table 3:** Description of original data and SMOTE-processed data.

**Dataset**	**Minority class**	**Majority class**	**Samples in total**
Training	260	4,182	4,442
Training-smote	4,182	4,182	8,364
Testing	112	1,792	1,904
Testing-smote	1,792	1,792	3,584

**Figure 2:**
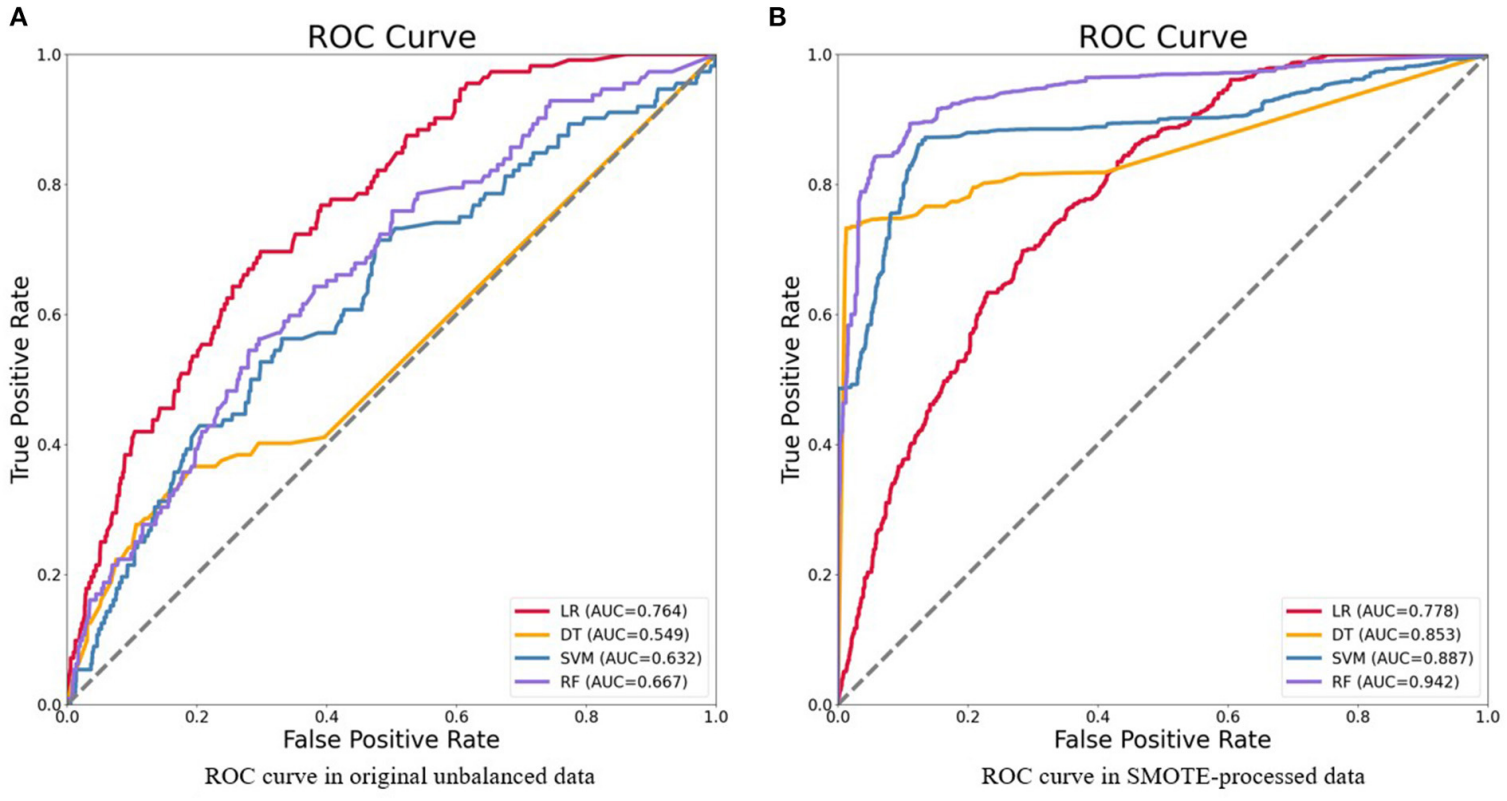
Receiver operating characteristics (ROC) curve of four models for the prediction of HIV in original unbalanced data **(A)** and SMOTE-processed data **(B)**. LR, logistic regression; DT, decision tree; SVM, support vector machines; RF, random forest.

In the second stage, we developed models by using the training data. In reference to Table 4, we summarized the performance of prediction models by validating in testing data. We can see that the only advisable indicator of these models was accuracy (>0.934). However, the results of other indicators were not great that recall ranged from a low of 0.009 to a high of 0.045 and F-1 ranged from a low of 0.016 to a high of 0.074. In the third stage, we also developed models by taking the training-smote data. Compared to the prediction effects of models in the original dataset, the performances of four models in SMOTE-processed data were much better, as shown in Table 5. The accuracy calculated by LR, DT, SVM, and RF was 0.702, 0.852, 0.811, and 0.871, respectively; the precision was 0.690, 0.954, 0.906, and 0.960, respectively; the recall was 0.733, 0.741, 0.695, and 0.755, respectively; the F-measure was 0.711, 0.834, 0.787, and 0.858, respectively; the AUC value was 0.778, 0.853, 0.887, and 0.942, respectively. ROC curves of four algorithms in two situations were shown in Figure 2.

**Table 4:** Results of classification models in original unbalanced data.

**Models**	**Accuracy**	**Precision**	**Recall**	**F-measure**	**AUC**
LR	0.941	0.500	0.009	0.018	0.764
DT	0.934	0.208	0.045	0.074	0.549
SVM	0.935	0.071	0.009	0.016	0.632
RF	0.934	0.118	0.018	0.031	0.667

**Table 5:** Results of classification models in SMOTE-processed data.

**Models**	**Accuracy**	**Precision**	**Recall**	**F-measure**	**AUC**
LR	0.702	0.690	0.733	0.711	0.778
DT	0.852	0.954	0.741	0.834	0.853
SVM	0.811	0.906	0.695	0.787	0.887
RF	0.871	0.960	0.775	0.858	0.942

### Prospective validation

**Figure 3:**
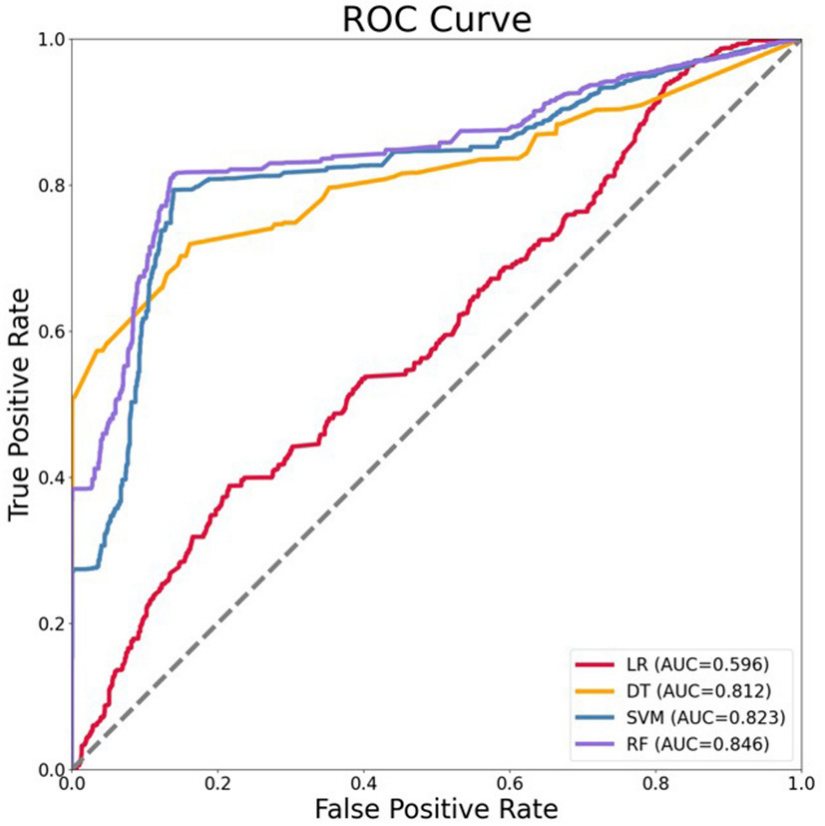
Receiver operating characteristics (ROC) curve of four models for the prediction of HIV in prospective validation data. LR, logistic regression; DT, decision tree; SVM, support vector machines; RF, random forest.

According to the results of the models above, we used the prospective validation data to further verify the extensibility of models in this stage. The basic characteristics of variables in 2020 data were shown in Table 2. ROC curves of four algorithms were shown in Figure 3. The final results showed that RF model also exhibits better performance compared with LR, DT, SVM (with the AUC of 0.596, 0.812, 0.823, and 0.846, respectively). Compared with the AUC of the RF model in the internal testing set, we found that the AUC of the RF model in the prospective validation set decreased by 0.096.

## Discussion

MSM are one of the high-risk groups because they are susceptible to infection after engaging in unprotected anal sex (27). An updated systematic review and meta-analysis revealed that the overall HIV incidence for multiple periods among MSM in China was a rising trend, which pooled separately from 2005 to 2008 (3.24/100 PY), 2009 to 2011 (5.29/100 PY), and 2012 to 2014 (5.50/100 PY) (28). Failure to test and receive antiretroviral treatment in time will lead to the progression of diseases and ultimately to the development of AIDS, so MSM has been identified as a priority population for HIV prevention and control interventions in China (29). In response to the fact that the detection rate of MSM population is lower than the first of UNAIDS' 90-90-90 targets, we need to find a fairly high accuracy model for the prediction of HIV status.

To our knowledge, this is the first study to apply machine learning on AIDS sentinel surveillance data to predict HIV infection among MSM in China. The predictive model by machine learning can distinguish between high and low risks. As long as an individual has a predictive value of one, he or she is considered to be at high risk for HIV infection and could benefit from early additional screening and diagnosis (30). In the present study, we examined whether machine learning algorithms provide more accurate prediction models for HIV infection in MSM than the conventional logistic regression model.

In the beginning, the predictors selected for this study were independently associated with HIV infection, including important sexual behavior factors, such as condom use in the latest homosexual anal sex (OR = 0.30, 95% CI: 0.24–0.37), frequency of condom use was every time (OR = 0.19, 95% CI: 0.13–0.29), and diagnosed with sexually transmitted diseases (OR = 1.45, 95% CI: 1.02–2.26). These above variables were generally reported in recent HIV-related studies of behavioral risk factors (31, 32).

Our study shows that the approach of machine learning is feasible and fairly high accuracy. We compared LR, DT, SVM, and RF, and accuracy, precision, recall, F-measure, and AUC value of each model were analyzed. In unbalanced original data, we found that only the indicator of accuracy was acceptable and the other indicators were poor. However, using this metric alone is not meaningful because class distributions that are highly skewed tend to bias the results of machine learning algorithms (33). Even if all cases are predicted to be negative, the accuracy of the model is also more than 90%, but the precision and recall are both 0 (15). Therefore, it is not enough to represent great classifiers in terms of high accuracy value. Then, the comprehensive evaluation indices of machine learning models in SMOTE-processed data were better than traditional logistic regression model, in which the RF model performed best (accuracy = 0.871, precision = 0.960, recall = 0.775, F-measure = 0.858, AUC = 0.942). In addition, the RF model also performed well when the optimal model was prospectively validated with 2020 data (AUC = 0.846). The above results indicate that advanced methods of machine learning can be used to develop models with higher prediction accuracy, where the performance of RF is satisfactory to predict HIV status among MSM in China.

Previous studies have also provided evidence of using machine learning algorithms in predicting HIV infection. Krakower et al. (34) developed and validated multiple machine learning models to identify potential HIV pre-exposure prophylaxis (PrEP) candidates by using electronic health records containing 180 potential predictors from an ambulatory practice in Massachusetts in America, found that the best-performing algorithm was obtained with the least absolute shrinkage and selection operator (LASSO) (AUC = 0.86). In a similar setting in California, Marcus et al. (35) used 81 electronic health record variables to identify PrEP candidates by machine learning and demonstrated improved ability to predict incident HIV with inclusion of multiple data domains compared with simpler algorithms that based on MSM status and STI positivity (AUC = 0.86). In Denmark, Ahlstrom et al. (36) applied various machine learning methods in electronic registry data to predict HIV status and found that the RF algorithm also performed slightly better (AUC = 0.89). More recently, Bao et al. (37) developed four machine learning models and evaluated their performance in predicting HIV diagnosis based on a cohort of MSM in Australia, and he proposed that Machine learning approaches outperformed the multivariable logistic regression model, with the gradient boosting machine achieving the highest performance (AUC = 0.76). Our study complements these machine learning studies applied to HIV infection prediction, all of which effectively illustrate that machine learning can be used as an effective method for detecting HIV infection among MSM.

There were several limitations to this study. First, although the questionnaire information was collected through individual interviews between survey subjects and health professionals, some of this occurred in the past, which is subjected to the recall bias. Moreover, the questionnaire needs to be further supplemented due to the absence of some behavioral characteristics (e.g., the number of sexual partners, sex role of accessor/recipient) (38). Second, we only employed the three most commonly-used machine learning algorithms for classification results prediction, so other useful models and methods can be explored in future research, including natural language processing in unstructured data (39). Third, since the research subjects selected for models building came from only Zhejiang province, further exploration is needed in generalizing the optimal model to the whole country and making it universally applicable. Fourth, machine learning for effectively avoiding overfitting is a crucial strategy (40). Our models may have the problem of overfitting and should address it by regularization and penalization of model complexity (41).

In conclusion, the study shows that machine learning has an advantage over traditional models in predicting HIV infection among MSM and the RF has a superior performance. In particular, SMOTE technology helps models to achieve better performance when facing unbalanced data. Within an increase in HIV incidence among MSM, even other high-risk populations, it is expected that prediction models based on machine learning for HIV infection can be an important direction to discriminate whether they are at high-risk for HIV acquisition to be provided with timely interventional treatment. Furthermore, additional researches are needed to further optimize these algorithms, expand useful models to the entire country, and evaluate their usefulness and effects of them on HIV prevention.

## Data availability statement

The original contributions presented in the study are included in the article/Supplementary material, further inquiries can be directed to the corresponding author/s.

## Author contributions

JH, SJ, and CW came up with the original idea. JH and JL participated in the research design and provided research methods. WC, JJ, YX, JY, XZ, and CC completed the data collection and improved the manuscript. JH performed the data analysis and drafted the manuscript. All authors contributed to the article and approved the submitted version.

## Funding

This study was supported by the National Key Research and Development Project of China (2021ZD0110400 and 2018AAA0101900), National Natural Science Foundation of China (U19B2042), Program of Zhejiiang Province Science and Technology (2022C01044), The University Synergy Innovation Program of Anhui Province (GXXT-2021-004), Zhejiang Lab (2021KE0AC02), Academy of Social Governance Zhejiang University, Fundamental Research Funds for the Central Universities (226-2022-00064), Artificial Intelligence Research Foundation of Baidu Inc., Program of ZJU and Tongdun Joint Research Lab.

## Conflict of interest

The authors declare that the research was conducted in the absence of any commercial or financial relationships that could be construed as a potential conflict of interest.

## Publisher's note

All claims expressed in this article are solely those of the authors and do not necessarily represent those of their affiliated organizations, or those of the publisher, the editors and the reviewers. Any product that may be evaluated in this article, or claim that may be made by its manufacturer, is not guaranteed or endorsed by the publisher.
