# Supplementary material for: Application of machine learning algorithms in predicting HIV infection among men who have sex with men: Model development and validation
Source: Front Public Health. 2022 Aug 25;10:967681. doi: 10.3389/fpubh.2022.967681 (PMC9452878; doi:10.3389/fpubh.2022.967681)
Supplement: Supplementary file 1 [file Table_1.docx]

**Supplementary materials**

Table 1 Key hyperparameters of each model

| Model | Hyperparameters | Original data | SMOTE data | Range |
| --- | --- | --- | --- | --- |
| LR | C | 0.1 | 0.5 | [0.1, 1, 0.1] |
| DT | max_depth | 25 | 29 | [1, 30, 1] |
|  | min_samples_spilt | 3 | 4 | [1, 10, 1] |
| SVM | C | 1 | 1 | [0.1, 1, 0.1] |
|  | gamma | 0.4 | 0.7 | [0.1, 1, 0.1] |
| RF | n_estimators | 150 | 170 | [10, 200, 10] |
|  | max_depth | 15 | 19 | [1, 30, 1] |
